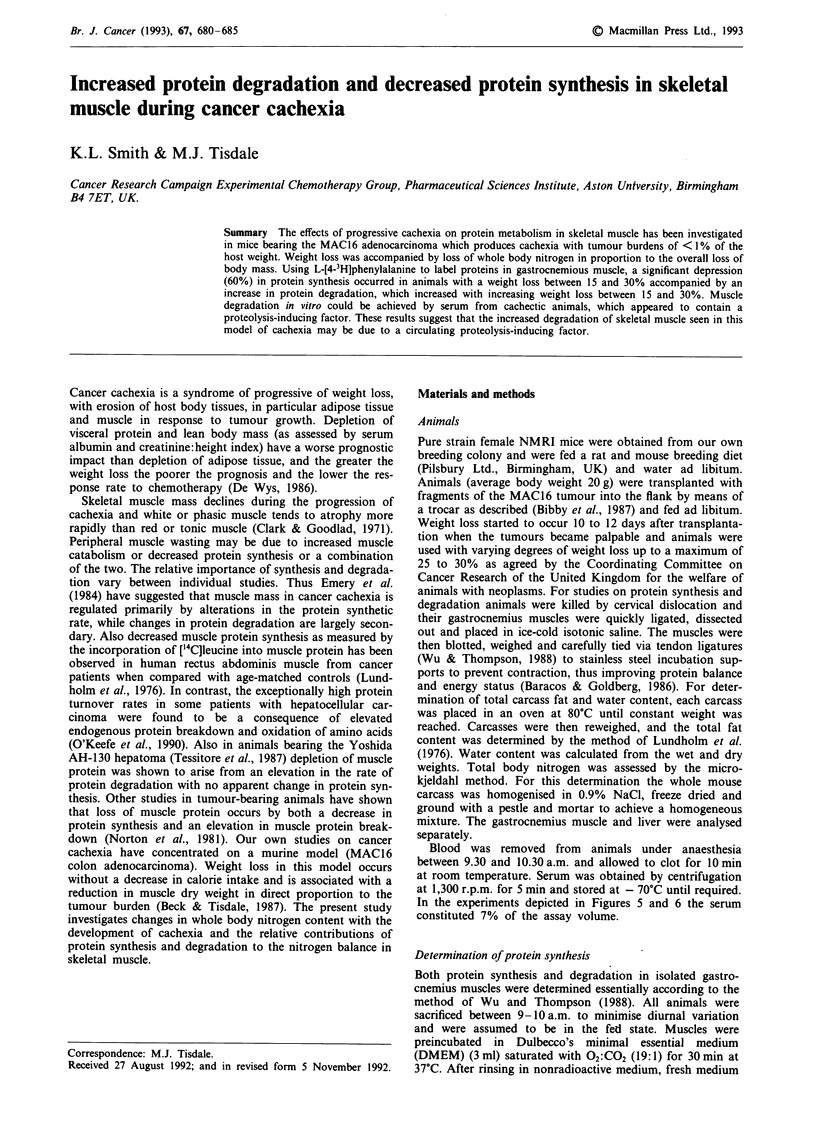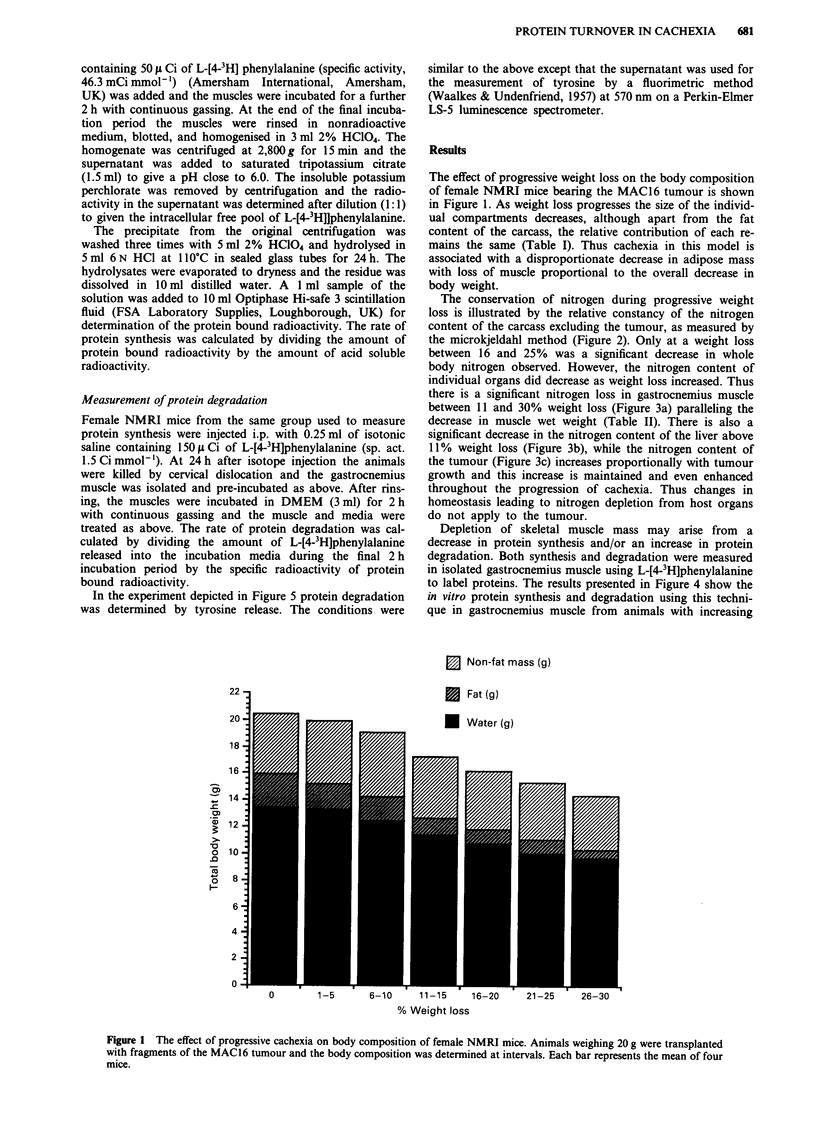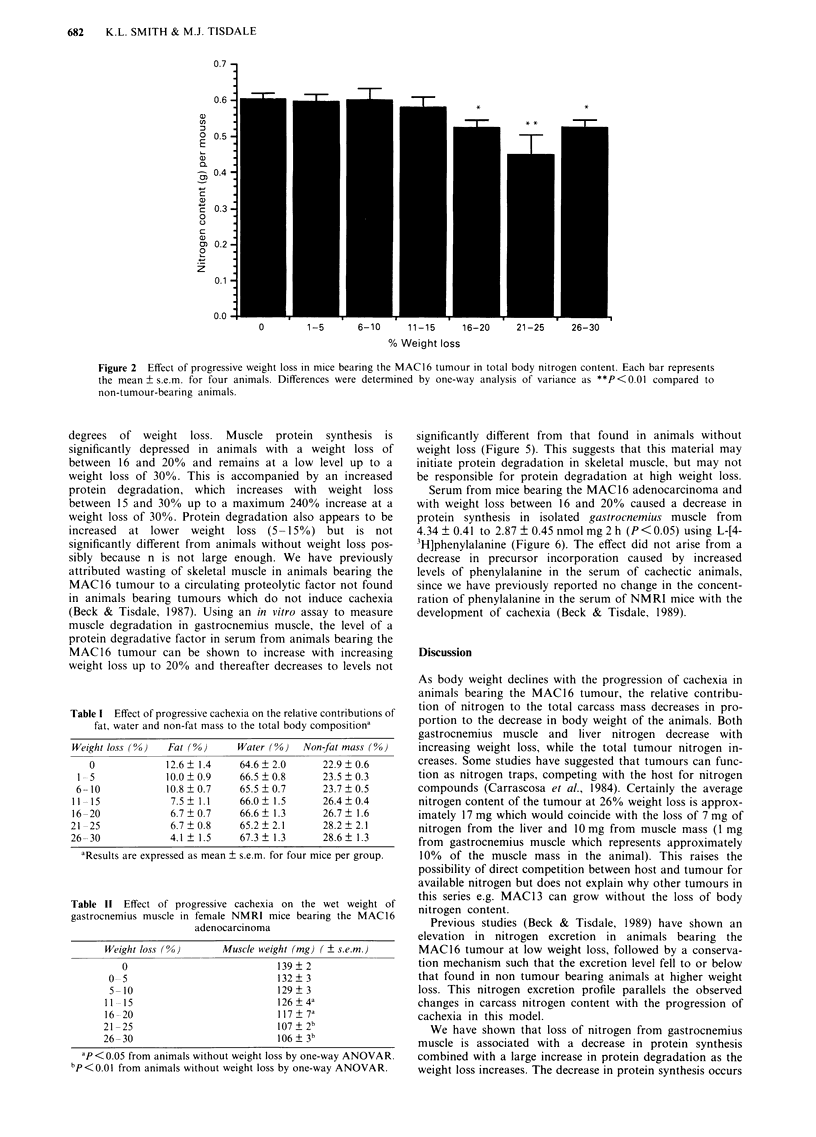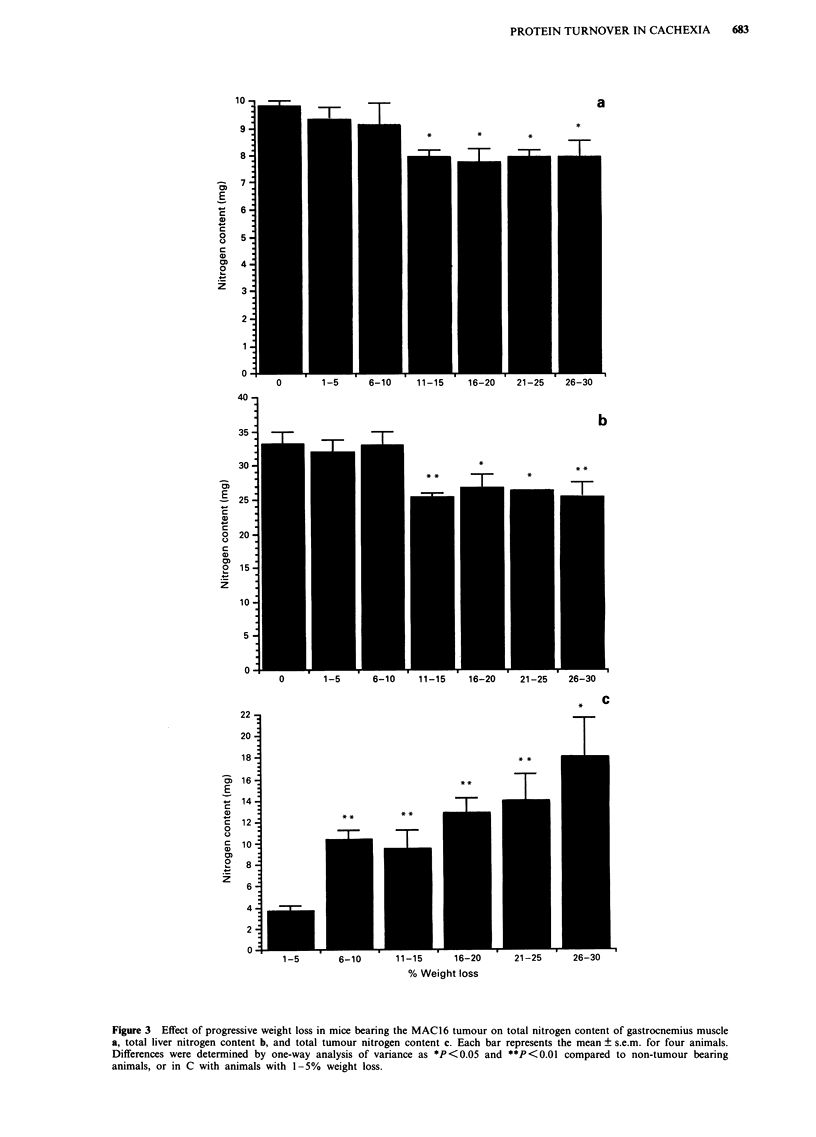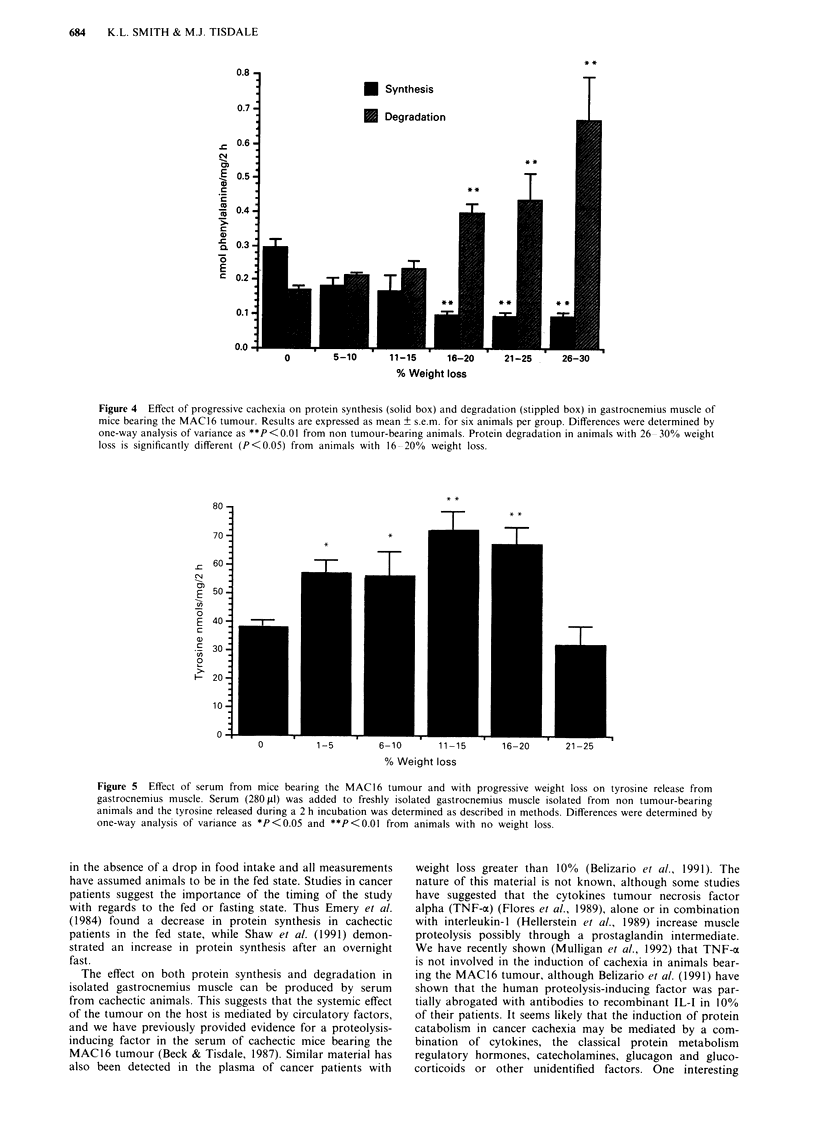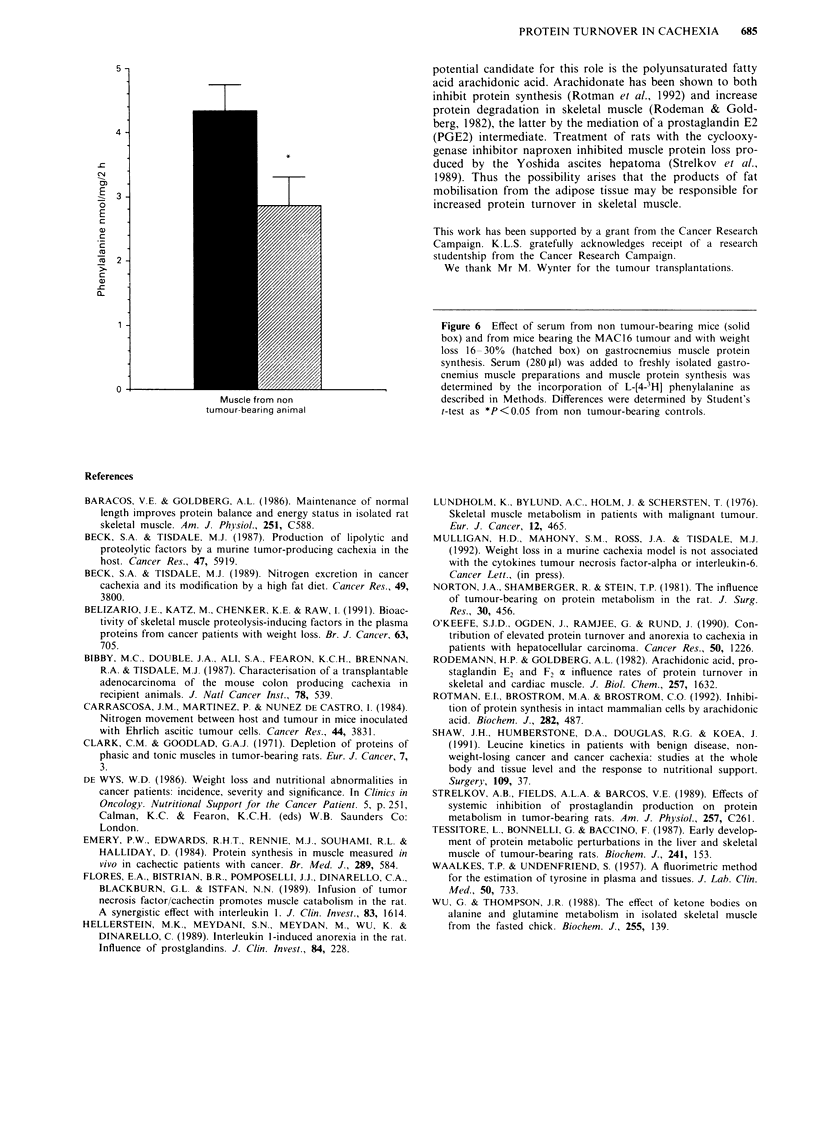# Increased protein degradation and decreased protein synthesis in skeletal muscle during cancer cachexia.

**DOI:** 10.1038/bjc.1993.126

**Published:** 1993-04

**Authors:** K. L. Smith, M. J. Tisdale

**Affiliations:** Cancer Research Campaign Experimental Chemotherapy Group, Pharmaceutical Sciences Institute, Aston University, Birmingham, UK.

## Abstract

The effects of progressive cachexia on protein metabolism in skeletal muscle has been investigated in mice bearing the MAC16 adenocarcinoma which produces cachexia with tumour burdens of < 1% of the host weight. Weight loss was accompanied by loss of whole body nitrogen in proportion to the overall loss of body mass. Using L-[4-3H]phenylalanine to label proteins in gastrocnemius muscle, a significant depression (60%) in protein synthesis occurred in animals with a weight loss between 15 and 30% accompanied by an increase in protein degradation, which increased with increasing weight loss between 15 and 30%. Muscle degradation in vitro could be achieved by serum from cachectic animals, which appeared to contain a proteolysis-inducing factor. These results suggest that the increased degradation of skeletal muscle seen in this model of cachexia may be due to a circulating proteolysis-inducing factor.


Br. J. Cancer (1993), 67, 680-685 © Macmillan Press Ltd., 1993

### Increased protein degradation and decreased protein synthesis in skeletal muscle during cancer cachexia

K.L. Smith & M.J. Tisdale

*Cancer Research Campaign Experimental Chemotherapy Group, Pharmaceutical Sciences Institute, Aston University, Birmingham B4 7ET, UK.*

**Summary** The effects of progressive cachexia on protein metabolism in skeletal muscle has been investigated in mice bearing the MAC16 adenocarcinoma which produces cachexia with tumour burdens of <1% of the host weight. Weight loss was accompanied by loss of whole body nitrogen in proportion to the overall loss of body mass. Using L-[4-$^{3}$H]phenylalanine to label proteins in gastrocnemious muscle, a significant depression (60%) in protein synthesis occurred in animals with a weight loss between 15 and 30% accompanied by an increase in protein degradation, which increased with increasing weight loss between 15 and 30%. Muscle degradation *in vitro* could be achieved by serum from cachectic animals, which appeared to contain a proteolysis-inducing factor. These results suggest that the increased degradation of skeletal muscle seen in this model of cachexia may be due to a circulating proteolysis-inducing factor.

Cancer cachexia is a syndrome of progressive of weight loss, with erosion of host body tissues, in particular adipose tissue and muscle in response to tumour growth. Depletion of visceral protein and lean body mass (as assessed by serum albumin and creatinine:height index) have a worse prognostic impact than depletion of adipose tissue, and the greater the weight loss the poorer the prognosis and the lower the response rate to chemotherapy (De Wys, 1986).

Skeletal muscle mass declines during the progression of cachexia and white or phasic muscle tends to atrophy more rapidly than red or tonic muscle (Clark & Goodlad, 1971). Peripheral muscle wasting may be due to increased muscle catabolism or decreased protein synthesis or a combination of the two. The relative importance of synthesis and degradation vary between individual studies. Thus Emery *et al.* (1984) have suggested that muscle mass in cancer cachexia is regulated primarily by alterations in the protein synthetic rate, while changes in protein degradation are largely secondary. Also decreased muscle protein synthesis as measured by the incorporation of [$^{14}$C]leucine into muscle protein has been observed in human rectus abdominis muscle from cancer patients when compared with age-matched controls (Lundholm *et al.*, 1976). In contrast, the exceptionally high protein turnover rates in some patients with hepatocellular carcinoma were found to be a consequence of elevated endogenous protein breakdown and oxidation of amino acids (O'Keefe *et al.*, 1990). Also in animals bearing the Yoshida AH-130 hepatoma (Tessitore *et al.*, 1987) depletion of muscle protein was shown to arise from an elevation in the rate of protein degradation with no apparent change in protein synthesis. Other studies in tumour-bearing animals have shown that loss of muscle protein occurs by both a decrease in protein synthesis and an elevation in muscle protein breakdown (Norton *et al.*, 1981). Our own studies on cancer cachexia have concentrated on a murine model (MAC16 colon adenocarcinoma). Weight loss in this model occurs without a decrease in calorie intake and is associated with a reduction in muscle dry weight in direct proportion to the tumour burden (Beck & Tisdale, 1987). The present study investigates changes in whole body nitrogen content with the development of cachexia and the relative contributions of protein synthesis and degradation to the nitrogen balance in skeletal muscle.

Correspondence: M.J. Tisdale.
Received 27 August 1992; and in revised form 5 November 1992.

### Materials and methods

### *Animals*

Pure strain female NMRI mice were obtained from our own breeding colony and were fed a rat and mouse breeding diet (Pilsbury Ltd., Birmingham, UK) and water ad libitum. Animals (average body weight 20 g) were transplanted with fragments of the MAC16 tumour into the flank by means of a trocar as described (Bibby *et al.*, 1987) and fed ad libitum. Weight loss started to occur 10 to 12 days after transplantation when the tumours became palpable and animals were used with varying degrees of weight loss up to a maximum of 25 to 30% as agreed by the Coordinating Committee on Cancer Research of the United Kingdom for the welfare of animals with neoplasms. For studies on protein synthesis and degradation animals were killed by cervical dislocation and their gastrocnemius muscles were quickly ligated, dissected out and placed in ice-cold isotonic saline. The muscles were then blotted, weighed and carefully tied via tendon ligatures (Wu & Thompson, 1988) to stainless steel incubation supports to prevent contraction, thus improving protein balance and energy status (Baracos & Goldberg, 1986). For determination of total carcass fat and water content, each carcass was placed in an oven at 80°C until constant weight was reached. Carcasses were then reweighed, and the total fat content was determined by the method of Lundholm *et al.* (1976). Water content was calculated from the wet and dry weights. Total body nitrogen was assessed by the microkjeldahl method. For this determination the whole mouse carcass was homogenised in 0.9% NaCl, freeze dried and ground with a pestle and mortar to achieve a homogeneous mixture. The gastrocnemius muscle and liver were analysed separately.

Blood was removed from animals under anaesthesia between 9.30 and 10.30 a.m. and allowed to clot for 10 min at room temperature. Serum was obtained by centrifugation at 1,300 r.p.m. for 5 min and stored at − 70°C until required. In the experiments depicted in Figures 5 and 6 the serum constituted 7% of the assay volume.

### *Determination of protein synthesis*

Both protein synthesis and degradation in isolated gastrocnemius muscles were determined essentially according to the method of Wu and Thompson (1988). All animals were sacrificed between 9-10 a.m. to minimise diurnal variation and were assumed to be in the fed state. Muscles were preincubated in Dulbecco's minimal essential medium (DMEM) (3 ml) saturated with $O_2$:$CO_2$ (19:1) for 30 min at 37°C. After rinsing in nonradioactive medium, fresh medium containing 50 $\mu$Ci of L-[4-$^3$H] phenylalanine (specific activity, 46.3 mCi mmol$^{-1}$) (Amersham International, Amersham, UK) was added and the muscles were incubated for a further 2 h with continuous gassing. At the end of the final incubation period the muscles were rinsed in nonradioactive medium, blotted, and homogenised in 3 ml 2% $HClO_4$. The homogenate was centrifuged at 2,800 *g* for 15 min and the supernatant was added to saturated tripotassium citrate (1.5 ml) to give a pH close to 6.0. The insoluble potassium perchlorate was removed by centrifugation and the radioactivity in the supernatant was determined after dilution (1:1) to given the intracellular free pool of L-[4-$^3$H]]phenylalanine.

The precipitate from the original centrifugation was washed three times with 5 ml 2% $HClO_4$ and hydrolysed in 5 ml 6 N HCl at 110°C in sealed glass tubes for 24 h. The hydrolysates were evaporated to dryness and the residue was dissolved in 10 ml distilled water. A 1 ml sample of the solution was added to 10 ml Optiphase Hi-safe 3 scintillation fluid (FSA Laboratory Supplies, Loughborough, UK) for determination of the protein bound radioactivity. The rate of protein synthesis was calculated by dividing the amount of protein bound radioactivity by the amount of acid soluble radioactivity.

### *Measurement of protein degradation*

Female NMRI mice from the same group used to measure protein synthesis were injected i.p. with 0.25 ml of isotonic saline containing 150 $\mu$Ci of L-[4-$^3$H]phenylalanine (sp. act. 1.5 Ci mmol$^{-1}$). At 24 h after isotope injection the animals were killed by cervical dislocation and the gastrocnemius muscle was isolated and pre-incubated as above. After rinsing, the muscles were incubated in DMEM (3 ml) for 2 h with continuous gassing and the muscle and media were treated as above. The rate of protein degradation was calculated by dividing the amount of L-[4-$^3$H]phenylalanine released into the incubation media during the final 2 h incubation period by the specific radioactivity of protein bound radioactivity.

In the experiment depicted in Figure 5 protein degradation was determined by tyrosine release. The conditions were similar to the above except that the supernatant was used for the measurement of tyrosine by a fluorimetric method (Waalkes & Undenfriend, 1957) at 570 nm on a Perkin-Elmer LS-5 luminescence spectrometer.

### Results

The effect of progressive weight loss on the body composition of female NMRI mice bearing the MAC16 tumour is shown in Figure 1. As weight loss progresses the size of the individual compartments decreases, although apart from the fat content of the carcass, the relative contribution of each remains the same (Table I). Thus cachexia in this model is associated with a disproportionate decrease in adipose mass with loss of muscle proportional to the overall decrease in body weight.

The conservation of nitrogen during progressive weight loss is illustrated by the relative constancy of the nitrogen content of the carcass excluding the tumour, as measured by the microkjeldahl method (Figure 2). Only at a weight loss between 16 and 25% was a significant decrease in whole body nitrogen observed. However, the nitrogen content of individual organs did decrease as weight loss increased. Thus there is a significant nitrogen loss in gastrocnemius muscle between 11 and 30% weight loss (Figure 3a) paralleling the decrease in muscle wet weight (Table II). There is also a significant decrease in the nitrogen content of the liver above 11% weight loss (Figure 3b), while the nitrogen content of the tumour (Figure 3c) increases proportionally with tumour growth and this increase is maintained and even enhanced throughout the progression of cachexia. Thus changes in homeostasis leading to nitrogen depletion from host organs do not apply to the tumour.

Depletion of skeletal muscle mass may arise from a decrease in protein synthesis and/or an increase in protein degradation. Both synthesis and degradation were measured in isolated gastrocnemius muscle using L-[4-$^3$H]phenylalanine to label proteins. The results presented in Figure 4 show the *in vitro* protein synthesis and degradation using this technique in gastrocnemius muscle from animals with increasing

**Figure 1** The effect of progressive cachexia on body composition of female NMRI mice. Animals weighing 20 g were transplanted with fragments of the MAC16 tumour and the body composition was determined at intervals. Each bar represents the mean of four mice.

**Figure 2** Effect of progressive weight loss in mice bearing the MAC16 tumour in total body nitrogen content. Each bar represents the mean ± s.e.m. for four animals. Differences were determined by one-way analysis of variance as $^{**}P<0.01$ compared to non-tumour-bearing animals.

degrees of weight loss. Muscle protein synthesis is significantly depressed in animals with a weight loss of between 16 and 20% and remains at a low level up to a weight loss of 30%. This is accompanied by an increased protein degradation, which increases with weight loss between 15 and 30% up to a maximum 240% increase at a weight loss of 30%. Protein degradation also appears to be increased at lower weight loss (5–15%) but is not significantly different from animals without weight loss possibly because n is not large enough. We have previously attributed wasting of skeletal muscle in animals bearing the MAC16 tumour to a circulating proteolytic factor not found in animals bearing tumours which do not induce cachexia (Beck & Tisdale, 1987). Using an *in vitro* assay to measure muscle degradation in gastrocnemius muscle, the level of a protein degradative factor in serum from animals bearing the MAC16 tumour can be shown to increase with increasing weight loss up to 20% and thereafter decreases to levels not significantly different from that found in animals without weight loss (Figure 5). This suggests that this material may initiate protein degradation in skeletal muscle, but may not be responsible for protein degradation at high weight loss.

Serum from mice bearing the MAC16 adenocarcinoma and with weight loss between 16 and 20% caused a decrease in protein synthesis in isolated *gastrocnemius* muscle from 4.34 ± 0.41 to 2.87 ± 0.45 nmol mg 2 h ($P<0.05$) using L-[4-$^3$H]phenylalanine (Figure 6). The effect did not arise from a decrease in precursor incorporation caused by increased levels of phenylalanine in the serum of cachectic animals, since we have previously reported no change in the concentration of phenylalanine in the serum of NMRI mice with the development of cachexia (Beck & Tisdale, 1989).

**Table I** Effect of progressive cachexia on the relative contributions of fat, water and non-fat mass to the total body composition[a]

| *Weight loss (%)* | *Fat (%)* | *Water (%)* | *Non-fat mass (%)* |
|---|---|---|---|
| 0 | 12.6 ± 1.4 | 64.6 ± 2.0 | 22.9 ± 0.6 |
| 1–5 | 10.0 ± 0.9 | 66.5 ± 0.8 | 23.5 ± 0.3 |
| 6–10 | 10.8 ± 0.7 | 65.5 ± 0.7 | 23.7 ± 0.5 |
| 11–15 | 7.5 ± 1.1 | 66.0 ± 1.5 | 26.4 ± 0.4 |
| 16–20 | 6.7 ± 0.7 | 66.6 ± 1.3 | 26.7 ± 1.6 |
| 21–25 | 6.7 ± 0.8 | 65.2 ± 2.1 | 28.2 ± 2.1 |
| 26–30 | 4.1 ± 1.5 | 67.3 ± 1.3 | 28.6 ± 1.3 |

[a]Results are expressed as mean ± s.e.m. for four mice per group.

**Table II** Effect of progressive cachexia on the wet weight of gastrocnemius muscle in female NMRI mice bearing the MAC16 adenocarcinoma

| *Weight loss (%)* | *Muscle weight (mg) (± s.e.m.)* |
|---|---|
| 0 | 139 ± 2 |
| 0–5 | 132 ± 3 |
| 5–10 | 129 ± 3 |
| 11–15 | 126 ± 4[a] |
| 16–20 | 117 ± 7[a] |
| 21–25 | 107 ± 2[b] |
| 26–30 | 106 ± 3[b] |

[a]$P<0.05$ from animals without weight loss by one-way ANOVAR.
[b]$P<0.01$ from animals without weight loss by one-way ANOVAR.

### Discussion

As body weight declines with the progression of cachexia in animals bearing the MAC16 tumour, the relative contribution of nitrogen to the total carcass mass decreases in proportion to the decrease in body weight of the animals. Both gastrocnemius muscle and liver nitrogen decrease with increasing weight loss, while the total tumour nitrogen increases. Some studies have suggested that tumours can function as nitrogen traps, competing with the host for nitrogen compounds (Carrascosa *et al.*, 1984). Certainly the average nitrogen content of the tumour at 26% weight loss is approximately 17 mg which would coincide with the loss of 7 mg of nitrogen from the liver and 10 mg from muscle mass (1 mg from gastrocnemius muscle which represents approximately 10% of the muscle mass in the animal). This raises the possibility of direct competition between host and tumour for available nitrogen but does not explain why other tumours in this series e.g. MAC13 can grow without the loss of body nitrogen content.

Previous studies (Beck & Tisdale, 1989) have shown an elevation in nitrogen excretion in animals bearing the MAC16 tumour at low weight loss, followed by a conservation mechanism such that the excretion level fell to or below that found in non tumour bearing animals at higher weight loss. This nitrogen excretion profile parallels the observed changes in carcass nitrogen content with the progression of cachexia in this model.

We have shown that loss of nitrogen from gastrocnemius muscle is associated with a decrease in protein synthesis combined with a large increase in protein degradation as the weight loss increases. The decrease in protein synthesis occurs

**Figure 3** Effect of progressive weight loss in mice bearing the MAC16 tumour on total nitrogen content of gastrocnemius muscle **a**, total liver nitrogen content **b**, and total tumour nitrogen content **c**. Each bar represents the mean ± s.e.m. for four animals. Differences were determined by one-way analysis of variance as *$P < 0.05$ and **$P < 0.01$ compared to non-tumour bearing animals, or in C with animals with 1–5% weight loss.

**Figure 4** Effect of progressive cachexia on protein synthesis (solid box) and degradation (stippled box) in gastrocnemius muscle of mice bearing the MAC16 tumour. Results are expressed as mean ± s.e.m. for six animals per group. Differences were determined by one-way analysis of variance as $^{**}P < 0.01$ from non tumour-bearing animals. Protein degradation in animals with 26–30% weight loss is significantly different ($P < 0.05$) from animals with 16–20% weight loss.

**Figure 5** Effect of serum from mice bearing the MAC16 tumour and with progressive weight loss on tyrosine release from gastrocnemius muscle. Serum (280 µl) was added to freshly isolated gastrocnemius muscle isolated from non tumour-bearing animals and the tyrosine released during a 2 h incubation was determined as described in methods. Differences were determined by one-way analysis of variance as $^{*}P < 0.05$ and $^{**}P < 0.01$ from animals with no weight loss.

in the absence of a drop in food intake and all measurements have assumed animals to be in the fed state. Studies in cancer patients suggest the importance of the timing of the study with regards to the fed or fasting state. Thus Emery *et al.* (1984) found a decrease in protein synthesis in cachectic patients in the fed state, while Shaw *et al.* (1991) demonstrated an increase in protein synthesis after an overnight fast.

The effect on both protein synthesis and degradation in isolated gastrocnemius muscle can be produced by serum from cachectic animals. This suggests that the systemic effect of the tumour on the host is mediated by circulatory factors, and we have previously provided evidence for a proteolysis-inducing factor in the serum of cachectic mice bearing the MAC16 tumour (Beck & Tisdale, 1987). Similar material has also been detected in the plasma of cancer patients with weight loss greater than 10% (Belizario *et al.*, 1991). The nature of this material is not known, although some studies have suggested that the cytokines tumour necrosis factor alpha (TNF-α) (Flores *et al.*, 1989), alone or in combination with interleukin-1 (Hellerstein *et al.*, 1989) increase muscle proteolysis possibly through a prostaglandin intermediate. We have recently shown (Mulligan *et al.*, 1992) that TNF-α is not involved in the induction of cachexia in animals bearing the MAC16 tumour, although Belizario *et al.* (1991) have shown that the human proteolysis-inducing factor was partially abrogated with antibodies to recombinant IL-I in 10% of their patients. It seems likely that the induction of protein catabolism in cancer cachexia may be mediated by a combination of cytokines, the classical protein metabolism regulatory hormones, catecholamines, glucagon and glucocorticoids or other unidentified factors. One interesting

**Figure 6** Effect of serum from non tumour-bearing mice (solid box) and from mice bearing the MAC16 tumour and with weight loss 16–30% (hatched box) on gastrocnemius muscle protein synthesis. Serum (280 μl) was added to freshly isolated gastrocnemius muscle preparations and muscle protein synthesis was determined by the incorporation of L-[4-$^3$H] phenylalanine as described in Methods. Differences were determined by Student's *t*-test as $*P<0.05$ from non tumour-bearing controls.

potential candidate for this role is the polyunsaturated fatty acid arachidonic acid. Arachidonate has been shown to both inhibit protein synthesis (Rotman *et al*., 1992) and increase protein degradation in skeletal muscle (Rodeman & Goldberg, 1982), the latter by the mediation of a prostaglandin E2 (PGE2) intermediate. Treatment of rats with the cyclooxygenase inhibitor naproxen inhibited muscle protein loss produced by the Yoshida ascites hepatoma (Strelkov *et al*., 1989). Thus the possibility arises that the products of fat mobilisation from the adipose tissue may be responsible for increased protein turnover in skeletal muscle.

This work has been supported by a grant from the Cancer Research Campaign. K.L.S. gratefully acknowledges receipt of a research studentship from the Cancer Research Campaign.

We thank Mr M. Wynter for the tumour transplantations.

### References

BARACOS, V.E. & GOLDBERG, A.L. (1986). Maintenance of normal length improves protein balance and energy status in isolated rat skeletal muscle. *Am. J. Physiol*., **251,** C588.

BECK, S.A. & TISDALE, M.J. (1987). Production of lipolytic and proteolytic factors by a murine tumor-producing cachexia in the host. *Cancer Res*., **47,** 5919.

BECK, S.A. & TISDALE, M.J. (1989). Nitrogen excretion in cancer cachexia and its modification by a high fat diet. *Cancer Res*., **49,** 3800.

BELIZARIO, J.E., KATZ, M., CHENKER, K.E. & RAW, I. (1991). Bioactivity of skeletal muscle proteolysis-inducing factors in the plasma proteins from cancer patients with weight loss. *Br. J. Cancer*, **63,** 705.

BIBBY, M.C., DOUBLE, J.A., ALI, S.A., FEARON, K.C.H., BRENNAN, R.A. & TISDALE, M.J. (1987). Characterisation of a transplantable adenocarcinoma of the mouse colon producing cachexia in recipient animals. *J. Natl Cancer Inst*., **78,** 539.

CARRASCOSA, J.M., MARTINEZ, P. & NUNEZ DE CASTRO, I. (1984). Nitrogen movement between host and tumour in mice inoculated with Ehrlich ascitic tumour cells. *Cancer Res*., **44,** 3831.

CLARK, C.M. & GOODLAD, G.A.J. (1971). Depletion of proteins of phasic and tonic muscles in tumor-bearing rats. *Eur. J. Cancer*, **7,** 3.

DE WYS, W.D. (1986). Weight loss and nutritional abnormalities in cancer patients: incidence, severity and significance. In *Clinics in Oncology. Nutritional Support for the Cancer Patient*. 5, p. 251, Calman, K.C. & Fearon, K.C.H. (eds) W.B. Saunders Co: London.

EMERY, P.W., EDWARDS, R.H.T., RENNIE, M.J., SOUHAMI, R.L. & HALLIDAY, D. (1984). Protein synthesis in muscle measured *in vivo* in cachectic patients with cancer. *Br. Med. J*., **289,** 584.

FLORES, E.A., BISTRIAN, B.R., POMPOSELLI, J.J., DINARELLO, C.A., BLACKBURN, G.L. & ISTFAN, N.N. (1989). Infusion of tumor necrosis factor/cachectin promotes muscle catabolism in the rat. A synergistic effect with interleukin 1. *J. Clin. Invest*., **83,** 1614.

HELLERSTEIN, M.K., MEYDANI, S.N., MEYDAN, M., WU, K. & DINARELLO, C. (1989). Interleukin 1-induced anorexia in the rat. Influence of prostglandins. *J. Clin. Invest*., **84,** 228.

LUNDHOLM, K., BYLUND, A.C., HOLM, J. & SCHERSTEN, T. (1976). Skeletal muscle metabolism in patients with malignant tumour. *Eur. J. Cancer*, **12,** 465.

MULLIGAN, H.D., MAHONY, S.M., ROSS, J.A. & TISDALE, M.J. (1992). Weight loss in a murine cachexia model is not associated with the cytokines tumour necrosis factor-alpha or interleukin-6. *Cancer Lett*., (in press).

NORTON, J.A., SHAMBERGER, R. & STEIN, T.P. (1981). The influence of tumour-bearing on protein metabolism in the rat. *J. Surg. Res*., **30,** 456.

O'KEEFE, S.J.D., OGDEN, J., RAMJEE, G. & RUND, J. (1990). Contribution of elevated protein turnover and anorexia to cachexia in patients with hepatocellular carcinoma. *Cancer Res*., **50,** 1226.

RODEMANN, H.P. & GOLDBERG, A.L. (1982). Arachidonic acid, prostaglandin $E_2$ and $F_2$ α influence rates of protein turnover in skeletal and cardiac muscle. *J. Biol. Chem*., **257,** 1632.

ROTMAN, E.I., BROSTROM, M.A. & BROSTROM, C.O. (1992). Inhibition of protein synthesis in intact mammalian cells by arachidonic acid. *Biochem. J*., **282,** 487.

SHAW, J.H., HUMBERSTONE, D.A., DOUGLAS, R.G. & KOEA, J. (1991). Leucine kinetics in patients with benign disease, non-weight-losing cancer and cancer cachexia: studies at the whole body and tissue level and the response to nutritional support. *Surgery*, **109,** 37.

STRELKOV, A.B., FIELDS, A.L.A. & BARCOS, V.E. (1989). Effects of systemic inhibition of prostaglandin production on protein metabolism in tumor-bearing rats. *Am. J. Physiol*., **257,** C261.

TESSITORE, L., BONNELLI, G. & BACCINO, F. (1987). Early development of protein metabolic perturbations in the liver and skeletal muscle of tumour-bearing rats. *Biochem. J*., **241,** 153.

WAALKES, T.P. & UNDENFRIEND, S. (1957). A fluorimetric method for the estimation of tyrosine in plasma and tissues. *J. Lab. Clin. Med*., **50,** 733.

WU, G. & THOMPSON, J.R. (1988). The effect of ketone bodies on alanine and glutamine metabolism in isolated skeletal muscle from the fasted chick. *Biochem. J*., **255,** 139.